# Nurses and midwives’ experiences of using non-pharmacological interventions for labour pain management: a qualitative study in Ghana

**DOI:** 10.1186/s12884-019-2311-x

**Published:** 2019-05-14

**Authors:** Edward Appiah Boateng, Linda Osaebea Kumi, Abigail Kusi-Amponsah Diji

**Affiliations:** 1 Department of Nursing, Kwame Nkrumah University of Science and Technology, Kumasi, Ghana; 2 37 Military Teaching Hospital, Accra, Ghana

**Keywords:** Labour pain relief method(s), Non-drug technique(s), Phenomenology, Maternity care provider(s), Africa

## Abstract

**Background:**

Non-pharmacological interventions hold promise in reducing labour pain, with minimal or no harm to the mother, foetus and the progress of labour and are simple and cost-effective. Yet their use has not been adequately explored in clinical settings, especially in sub-Saharan Africa.

**Methods:**

This was a descriptive phenomenological study. Fifteen (15) nurses and midwives working in labour wards of two hospitals in Ghana were interviewed. Data analysis was guided by the principles of coding by Bailey and the constant comparative approach to generate themes. Ethics approval was obtained from the 37 Military Teaching Hospital Institutional Review Board in Ghana.

**Results:**

Three major themes were identified that described the experiences of nurses and midwives regarding their use of non-pharmacological interventions in managing labour pain. These were familiarity with non-pharmacological interventions, perceived benefits of non-pharmacological interventions, and barriers to the use of non-pharmacological interventions in the management of labour pain.

**Conclusions:**

While some non-pharmacological pain management interventions were known and used by the nurses and midwives, they were not familiar with a good number of these interventions. Nurses and midwives perceived these interventions to be beneficial yet a number of barriers prevented easy utilisation.

**Electronic supplementary material:**

The online version of this article (10.1186/s12884-019-2311-x) contains supplementary material, which is available to authorized users.

## Background

Pain experienced during childbirth is a complex, multidimensional and subjective phenomenon [1] that is of great concern to both the expectant mother and the maternity healthcare professional. Although the experience of pain is inherent in the childbearing process [2], unrelieved labour pain can result in negative consequences for the expectant mother, her family, healthcare providers and healthcare systems at large. Apart from maternal consequences such as heightened stress, fear, depression, confusion [3], hypertension, hyperglycaemia, and constipation [4], unresolved labour pain can also compromise placental perfusion leading to asphyxia, late decelerations and its resultant foetal distress [5]. These create feelings of guilt and helplessness for the woman’s family [6] as well as a lack of confidence in the abilities of healthcare providers and systems in general [7].

A wide array of pharmacological and non-pharmacological interventions are available for the relief of labour pain [8, 9]. The use of pharmacological interventions for the management of labour pain has dominated the maternity care profession due to the efficacy of these measures in reducing pain in expectant mothers [9]. Nevertheless, these measures are relatively costly [10] and associated with adverse effects such as maternal nausea, vomiting, drowsiness [11], fever [12], headache, hypotension, urinary retention, nerve injury [13], and foetal respiratory depression [14]. Moreover, these drug techniques may be associated with increased risks of delayed second stage of labour, labour augmentation, instrumental delivery and caesarean section [15].

Non-pharmacological interventions hold promise in reducing labour pain, with minimal or no harm to the mother, foetus and the progress of labour and are simple and cost-effective [9]. These have the potential to reduce analgesic consumption during labour [8] and include massage, breathing techniques, positioning, hydrotherapy, music, guided imagery, acupressure, aromatherapy among others [16].

Nurses and midwives play an essential role in the management of labour pain and have been described by Lundgren and Dahlberg [17] as “anchored companions”. A professional nurse or midwife in Ghana is one who has successfully completed nursing or midwifery training in an accredited college or university and has registered with the Nursing and Midwifery Council of Ghana. The scope of practice of midwives, as set out by the Nursing and Midwifery Council (NMC) of Ghana, permits them to provide antenatal, intrapartum, postpartum, reproductive health and infant care, and some specialized care depending on their level of training. In Ghana, the midwife to women in fertility age (WIFA) ratio was reported to be 1: 907 [18]. Consequently, nurses are, sometimes, assigned to maternity care areas as a way of improving the ratios. Considering the central role nurses and midwives play in the childbirth process, this study sought to explore their experiences with using non-pharmacological interventions in labour pain management.

## Methods

### Study setting

This study was carried out in 2 hospitals in Ghana which we will call hospital A and B respectively for purposes of anonymity and confidentiality. Hospital A has a 400-bed capacity and an extended capacity of 600 beds in emergency situations. It serves as the government’s emergency response health facility and as a United Nation’s level IV medical facility in the West African sub-region that provides healthcare to soldiers and civilian workers. On the other hand, Hospital B has an ultra-modern 420-bed capacity and is currently being expanded to a 600-bed capacity. It serves as a regional hospital in the country. Both hospitals provide healthcare in varied specialities including obstetrics and gynaecology.

### Study design, participants and data collection procedures

This was a descriptive phenomenological study, and the report was guided by the Consolidated Criteria for Reporting Qualitative Research [19]. Fifteen (15) nurses and midwives who had worked on the labour wards of the two hospitals for a minimum period of 6 months were purposively sampled between May and October 2016. Following ethics approval, LOK approached eligible participants. The purpose of the study, its benefits, risks and the voluntary nature of participation were explained to them before obtaining their written informed consents. All eligible participants who were approached agreed to be part of the study. Meetings were subsequently arranged with participants who signed the consent form based on their availability and preferences. Characteristics of participants have been provided in Table 1 including their rank and years of clinical experience. Pseudonyms have been used to ensure the anonymity of participants. In-depth individual interviews were conducted using a semi-structured interview guide (Additional file 1) and were audio-recorded after obtaining participants’ permission. All individual interviews were conducted in the English language and in a quiet private room within each hospital, facilitated by one of the researchers (LOK). No other person was present in the room aside LOK and the participant during each interview session. There were no repeat interviews. The questions were framed based on the research objectives, existing literature, expert opinion and feedback from pre-testing with 5 midwives in a similar hospital. Field notes were written after each interview to detail observations that could not be captured via the audio recording. The duration of interviews sessions ranged between 27 and 56 min.

**Table 1:** Characteristics of Participants

SN	Participant	Rank	Years of clinical experience
1	Mary	Staff midwife (SM)	5 years
2	Rhoda	Staff midwife (SM)	6 years
3	Agartha	Senior Nursing Officer (SNO)	7 years
4	Constance	Staff midwife (SM)	1 year
5	Debbie	Staff midwife (SM)	3 years
6	Elizabeth	Staff midwife (SM)	3 years
7	Fatima	Senior Nursing Officer (SNO)	13 years
8	Hannah	Staff midwife (SM)	3 years
9	Joyce	Staff midwife (SM)	17 years
10	Lady	Staff midwife (SM)	17 years
11	Pat	Rotation Midwife (RM)	6 months
12	Suzzy	Staff midwife (SM)	3 years
13	Takyiwaa	Senior Staff Midwife (SSM)	6 years
14	Vicky	Senior Staff Midwife (SSM)	6 years
15	Wendy	Senior Staff Midwife (SSM)	6 years

### Data processing and analysis

The interviews were carefully listened to and transcribed verbatim after each session. In order to ensure non-distortion of collected data, the transcripts were returned to participants for feedback and/or corrections. Each participant-reviewed transcript was then read multiple times, grouped and organised throughout successive reads. The transcripts were coded by 3 researchers (LOK, AKA, EAB) to identify emerging themes which were discussed regularly throughout data collection by the entire research team. This was done to resolve differences and build consensus on identified themes that required further exploration in subsequent interviews.

Inductive data analysis was aided by NVivo version 12 and occurred concurrently with data collection. Data analysis was undertaken based on the principles of coding by Bailey [20] who describes two types of coding; initial and focused coding. With the initial coding, data was conceptualised and categorised through line-by-line coding. Secondly, focused coding involved grouping coded text into larger segments which comprised smaller segments. This comprised carefully identifying relationships between concepts that made up the main themes. Constant comparison between codes, memos and literature was maintained throughout the research work. After exploring the experiences of the nurses and midwives with using non-pharmacological labour pain management techniques, the results of the research study were compared with existing evidence in order to bring meaning to the research question. As suggested by O’Reilly and Parker [21], thematic data saturation was reached when no new themes emerged from the collected data in each of the hospitals.

### Trustworthiness of the study

A number of approaches were employed to enhance the trustworthiness of this study. Credibility was ensured by collecting data from two different hospitals to ensure comprehensive accounts on the experiences of nurses and midwives in Ghana [22]. All researchers were involved in the data coding and entire analysis process, meeting frequently to discuss and resolve differences on emerging themes to ensure investigator triangulation and comprehensive account of findings [23]. Inclusion of participants’ quotes to support researchers’ interpretations also added to the credibility of the study [24]. This approach also promoted confirmability by ensuring that interpretations were grounded in the data rather than mere viewpoints of the researchers [23]. Efforts have been made to describe the setting as well as key characteristics of participants while maintaining anonymity and confidentiality to ensure transferability of this study [23]. As explained above, member checks were also done by sending transcripts to participants for feedback and corrections [25].

### Ethical considerations

Ethics approval, with reference number 37MH-IRB IPN 078/2016, was obtained from the 37 Military Teaching Hospital Institutional Review Board. Participants were assured of anonymity, privacy and confidentiality of their responses as well as the voluntary nature of participation in this study.

## Results

Analysis of the data identified three (3) major thematic areas which described nurses and midwives’ experiences with the use of non-pharmacological interventions for labour pain management in the selected hospitals. These were familiarity with non-pharmacological interventions, benefits of non-pharmacological interventions, and barriers to the use of non-pharmacological interventions in the management of labour pain. These have been presented below, with relevant quotes from participants.

### Familiarity with non-pharmacological interventions

This theme described participants’ awareness and usage of non-pharmacological pain management techniques in midwifery practice to manage labour-associated pains. All participants provided an accurate explanation of non-pharmacological interventions for labour pain management.

*“They [non-pharmacological interventions] are ways of relieving labour pain in which no medications are used.” (Constance, SM)*

*“…using other means to relieve pain other than drugs” (Vicky, SSM)*

Participants reported learning about these methods from varied sources, including their midwifery or nursing training colleges, workplaces and workshops.

*“I learnt [about] most of these [non-pharmacological] methods on the wards especially the previous places I’ve worked... and I know there [is] a lot I will not even know of” (Joyce, SM)*

*“I went for this life-saving skills workshop some time ago and this non-pharmacological therapy was one of the topics” (Constance, SM)*

*“I was taught in school and my in-charge here encouraged me to use those methods” (Pat, RM).*

Participants were asked to provide examples of common non-pharmacological interventions they use in managing labour pain. All participants were able to list, at least, one non-pharmacological pain management method which they were aware of and used in their practice. The most frequently used methods as mentioned by participants were sacral massage, deep breathing exercises and diversional therapy.

*“You can also create the right environment by giving some diversional therapy ... you can ask the client to be breathing in and out, you can give a sacral massage as well.” (Hannah, SM)*

*“You [can] encourage patient during labour and give a sacral massage. You can involve the patient in a conversation which is something like a diversional therapy which will take her mind off the pain” (Elizabeth, RM)*

Other less often mentioned and presumably less frequently utilised methods were ambulation, cold shower, cold or warm compresses.

“*... ambulation …she should walk about; we usually tell the client that, she will feel the pain more if she lies on one side or at one place…” (Debbie, SM)*

*“…deep breathing exercise, encouraged to walk about, listen to the music of her choice or watch television…Then warm or cold towels can be used on the client’s abdomen, forehead or anywhere she feels comfortable especially when she is sweating.” (Joyce, SM)*

However, other types of non-pharmacological methods such as guided imagery, chromotherapy, acupuncture, transcutaneous electrical nerve stimulation (TENS) and homoeopathy were not mentioned by any of the participants, suggesting limited awareness or infrequent usage of these methods among our participants. Almost all the participants revealed that they use non-pharmacological methods on a daily basis in the management of labour pain when they were asked about the frequency of use of these methods.

*“Here [in the labour ward] we use these [non-pharmacological] methods every day, I mean very often” (Rhoda, SM)*

*“We actually use it here, the majority of our clients usually go through this method; the non-pharmacological therapy” (Elizabeth, RM)*

### Benefits of non-pharmacological interventions in the management of labour pain

The study also explored participants’ perspectives on benefits associated with using non-pharmacological interventions in managing labour pain. This theme describes the perceived benefits that participants recounted about non- pharmacological interventions that they were aware of. A large number of the participants mentioned that a key benefit among all the benefits was that the methods presented no side effects, as indicated in the quotes below:

*“There is no side effect. If you, for instance, come in labour and I allow you to watch a movie for instance, at least it will take your mind off the pain without causing any harm to you or your baby...” (Lady, SMO)*

*“… because there are no side effects and it can be used any time…” (Wendy, SSM)*

*“It [non-pharmacological interventions] really helps psychologically, comparing the pharmacological management, for instance, pethidine [which] sometimes affect the foetal heart rate” (Elizabeth, RM)*

Others also explained that using non-pharmacological interventions serves as a way of relaxing the woman in labour and making her more comfortable during the process. The ultimate effect is that labour progresses smoothly.

*“It makes the client relaxed …the clients usually remain calm and others remain comforted.” (Rhoda, SM)*

*“Involving the client in a conversation actually takes the client’s mind off the pain, somehow. You do that [have conversation] and you realise that even when the client is contracting, she does not really exhibit the pain as when there is no diversional therapy. It makes the client comfortable and decreases anxiety, makes them calm and makes the labour move on smoothly.” (Elizabeth, RM)*

*“It really calms the client, making her comfortable and takes her mind off the pain which thereby allows the labour to progress smoothly. It really helps and relaxes the client.” (Joyce, SM).*

Additionally, using non-pharmacological interventions was described as a means of fostering a good relationship between the nurse/midwife and the client, promoting client cooperation.

*“…it makes the client feel that you are close to her so whatever you ask her to do, naturally she does it.” (Lady, SMO)*

*“… and it was relieving because when you want to leave the client she goes like ‘auntie midwife, don’t go, please come back, come and do it again” (Agartha, SNO)*

Additionally, one participant mentioned that non-pharmacological interventions are easy to administer, cheap and that they are non-invasive.

*“…of course, since they are non-pharmacological, there are not many side-effects as compared to the pharmacological pain relievers. It’s the easiest pain relief you can give because you don’t have to use any invasive procedures, it has no side effect. It’s just physical, no invasion; nothing and it’s not sold.” (Vicky, SSM)*

### Barriers to the use of non-pharmacological interventions in the management of labour pain

Participants described impediments in their practice which discouraged and sometimes prevented them from using non-pharmacologic pain management interventions routinely in practice to assist expectant mothers in the delivery process. These reported barriers could be categorised into three – clinician-related, health system-related and client-centred.

Clinician-related barriers comprised perceptions and beliefs of nurses and midwives which hindered their frequent utilisation of non-pharmacological methods. Indeed, the previous section has presented perceptions about nurses and midwives on the benefits of utilising non-pharmacological interventions in the management of labour pain. Yet, a number of the nurses and midwives were of the view that non-pharmacological interventions do not actually relieve the pain. Such views were mainly held in comparison with pharmacological interventions, which they believe ensure a complete relief of pain.

*“If you are, for instance, doing sacral massage for the patient, that does not mean the pain is gone… the patient is only coping with the pain and the massage just takes her mind off the pain, that’s all, it does not really relieve the pain.” (Joyce, SM)*

“*I think that even though they feel relaxed the pain is still there; they can’t compare the relief they felt from that to the pharmacological therapy… but it does not take the pain away, that’s my own perception of it.” (Vicky, SSM)*

“*I think there are better ways to keep someone’s mind off the pain and with the breathing in the client still feels the pain, it is just that you the midwife keeps the client’s mind occupied with it but the pain still exists…but supposing you do the epidural, the client relaxes and is able to cooperate with you. The better way to relieve labour pain is with pharmacological therapy.” (Mary, SM)*

Consequently, nurses and midwives were not always ready to implement some of the aforementioned non-pharmacological interventions for women experiencing labour pain but preferred to administer pharmacologic therapy in most cases.

*“...if I did one [using non-pharmacological method] and she [client] said I should stop, if it were you would you have given her another [non-pharmacological] method?” (Mary, SM)*

Health system-related barriers were those that hindered utilisation of non-pharmacologic methods as a result of the structure of the healthcare system as well as the physical design of the labour wards. The overwhelming responsibilities of nurses and midwives as a result of the fewer numbers available within the healthcare facility per shift is one of such barriers. The implication is that the number of nurses and midwives available is not sufficient to effectively carry out these non-pharmacological interventions as some may require the constant presence of the attending maternity care provider to be effective.

*“Lack of staff, that is sometimes you have only two midwives on duty. These same midwives are to monitor clients to deliver, go to the theatre to resuscitate caesarean sectioned babies and write all that happens here as well as decontaminate.” (Mary, SM)*

*“It is time-consuming; you have to be present beside the client before you can administer it. It is not as if you are administering it and leaving, it’s only the pharmacological that you can administer and you can leave the client on her own.” (Agartha, SNO)*

*“If there are two women, who will I massage? And remember I’m the same midwife who will go and collect a baby at the [surgical] theatre and resuscitate, the same midwife managing an impromptu second stage, and not to mention all the documentation I have to do.” (Lady, SMO)*

Others also identified the setting of health facilities as a major barrier to the utilisation of some non-pharmacological interventions for managing labour pain. Some of the interventions could easily be administered by support persons of the woman in labour, including their partners. While their mere presence could be soothing, partners are also able to administer some of the non-pharmacological interventions when they are around.

*“…and sometimes in situations when the husbands are allowed to come into the labour ward; sometimes they touch them, give them massages and even the talk they have relieves them of their pain.” (Vicky, SSM)*

*“When the partner is involved it relieves the woman psychologically of pain since she feels belonged because the husband is around to caress her, give sacral massage [and] warm or cold drinks.” (Takyiwaa, SSM)*

However, there are no cubicles or private rooms for each client, making it inappropriate to have partners of each client in the labour ward. Other times, too, the preferred non-pharmacological intervention for one client may inconvenience other clients in the labour ward.

*“In our setting where we don’t have cubicles for nursing our clients…it becomes difficult when you have so many women in one room and you are to get a support person to come in, it’s not going to be comfortable” (Takyiwaa, SSM)*

*“In my ward, we have two labour beds so when you are involving one client in a conversation, the other client might not want to be disturbed…let’s say one client wants to listen to music and the other does not want to, it makes it difficult.” (Elizabeth, RM)*

The last but not the least barrier – client-centred – described preferences of clients which hindered the use of non-pharmacological methods in managing labour pain. Some midwives were of the opinion that client preferences, sometimes, serve as a barrier to the utilisation of non-pharmacological interventions. Although midwives may want to administer some of these interventions, the client may decide against them for various reasons.

*“Some patients can tell you ‘no, I don’t like this’ or ‘when you do the sacral massage it irritates me’ or ‘let me be, I think I’m OK without the sacral massage’”. (Elizabeth, RM)*

*“I remember I used the sacral massage on one client but she told me I should stop because it will make the baby’s eyes [become] red when it is born so I stopped.” (Mary, SM)*

This section has presented three main thematic areas with relevant supporting statements. In summary, midwives were generally familiar with non-pharmacologic interventions for managing labour pain as well as their benefits. However, a number of barriers that made the implementation of non-pharmacologic interventions challenging in the delivery of care in certain respects have also been presented.

## Discussion

The current study explored nurses and midwives’ experiences with using non-pharmacological interventions for labour pain management. Our study revealed that nurses and midwives were familiar with some non-pharmacologic approaches such as sacral massage, deep breathing, diversional therapy; and frequently use them in their practice to manage labour pain. The frequent usage of these methods has been reported in previous studies [26, 27] and can be attributed to the familiarity of the midwives with these approaches [28]. Other less frequently used methods like ambulation, cold shower, cold and warm compresses were also reported by nurses and midwives in the present study. While insufficient knowledge may account for the less frequent use of these methods, other factors such as inadequate human and material resources may have contributed to this observation, similar to what has been reported in other studies [29–31].

Although the nurses and midwives were aware of and used some non-pharmacologic labour pain management approaches, they were equally unaware of a good number of these techniques. Considering the critical role of nurses and midwives in the birthing process, this situation could limit the labour pain management options that expectant mothers have to choose from and may lead to sub-optimal labour pain relief. Non-pharmacological interventions such as water immersion [32], guided imagery, aromatherapy, chromotherapy, homoeopathy, acupuncture, transcutaneous electrical nerve stimulation (TENS), floral therapy, and energy-level support [33] have been shown to be successful in reducing labour pain in other settings. The use of these other non-pharmacological pain management interventions could be explored further in future studies.

Our study also revealed that the perceived benefits of non-pharmacological interventions for labour pain management contributed to its usage by the nurses and midwives. As reiterated in previous studies, participants perceived non-pharmacological interventions for pain management to be relatively cheap, non-invasive, easy to administer, associated with minimal or no side effects [34], reduce anxiety, promote comfort and help to form a trusting relationship between the attending midwife and the expectant mother [33, 35]. In spite of the reported use of non-pharmacologic methods, participating nurses and midwives also reported barriers which prevented them from using these methods in certain situations. Among them were their belief that such interventions do not really relieve pain as compared to pharmacological approaches, insufficient staff to deliver some of the time-consuming non-pharmacologic interventions, inappropriate layout of labour wards to ensure privacy and family participation, inconveniences to other clients and pregnant women’s refusal of some methods due to their perceived negative conceptions about some methods.

Participants’ belief that non-pharmacological methods “do not really” relieve pain is a demonstration of their insufficient knowledge on labour pain physiology and the mechanism of action of these non-pharmacologic techniques. Although non-pharmacological methods do not possess the same analgesic properties as pharmacologic techniques, they can reduce labour pain to an appreciable level [28, 36]. Considering that analgesics may be associated with untoward effects to both the labouring woman and her unborn baby [11, 14], the usage of non-pharmacological methods in addition to drug techniques hold promise in maximizing pain relief while minimizing side effects in the labour process [8, 37]. This is due to the reduced consumption of analgesics and their associated side effects when non-pharmacologic techniques are used to complement pharmacologic interventions. Moreover, several studies have demonstrated the effectiveness of non-pharmacological methods in reducing labour pain [28, 32, 38]. Exposure to and utilisation of diverse types of non-pharmacological methods could contribute to reducing the perception that these methods do not really relieve pain and this could be achieved through frequent training sessions on these lesser known methods. Indeed, our study revealed that nurses and midwives learn about non-pharmacological methods of labour pain management from various sources, including nursing and midwifery training colleges, wards and workshops. Emphasis on these methods during pre- and post-registration nursing and midwifery education would, therefore, build their knowledge-base while enhancing the utilisation of diverse forms of non-pharmacologic methods.

Similar to other studies [39, 40], insufficient staff served as a barrier to the utilisation of nonpharmacological methods in managing labour pain. Participants perceived some of the non-pharmacologic approaches to be time-consuming for the few attending maternity care providers to administer considering their heavy workload and increased client turnover. Excessive workload and increased client turnover invariably place a lot of stress on the few practising nurses and midwives, leading to staff burnout and impaired work efficiency [41, 42]. Incorporating teaching sessions on non-pharmacological interventions during ante-natal care could empower the expectant mother to support the few nurses and midwives in utilising these methods during labour management.

Another barrier that prevented the nurses and midwives from using non-pharmacologic labour pain relief strategies was the inappropriate design and layout of the delivery rooms which does not allow for privacy and family participation during the childbirth process. The process of labour is an intimate affair requiring the presence of supportive partners and family members without compromises on privacy [43, 44]. The poor state and layout of delivery rooms not only affects utilisation of non-pharmacological interventions for pain management but also often secludes the expectant mother from her loved ones and alone in the hands of healthcare professionals during the delivery process. This accounts for women delivering at home or with traditional birth attendants owing to their welcoming environment and company received during labour [45]. Designing labour wards to allow for the presence of family and significant others of the expectant mother will not only provide emotional support for the woman but also support with some non-pharmacological methods. In a setting where insufficient numbers of nurses and midwives serve as a barrier to the increased utilisation of these methods, ensuring the presence of a supportive family is an urgent requirement for effective utilisation of non-pharmacological methods.

﻿The inconveniences of some nonpharmacological methods to other clients were reported as one of the barriers to their usage at the labour wards. While some methods such as music listening or watching television serve as a form of distraction from pain for some women, other clients may prefer a noise-free environment during the delivery process. The international literature is clear about the advantages of ambience during labour [1, 46]. Creation of a conducive environment for the expectant mother can effectively reduce painful sensations during labour [47]. On this premise, it is important for maternity care providers to respect the individuality of each client and be prepared to modify the delivery environment to suit diverse clients [33].

Reported misconceptions by some clients also prevented nurses and midwives from using some of the non-pharmacologic pain relief techniques. For instance, one midwife shared an experience where a client perceived sacral massage to be associated with reddening of the newborn baby’s eyes. This suggests the need for client education on labour pain management during the antenatal period and reinforcement during the delivery process, so as to correct misconceptions. Special emphasis ought to be placed on the role of non-pharmacologic techniques as adjuncts to analgesics to improve labour pain relief and maximize birth outcomes without unnecessary medical interventions. Evidence suggests that many women opt for unnecessary interventions such as caesarean sections, instrumental delivery among others for fear of unrelieved labour pain [48, 49]. Considering that these interventions are not without complications such as excessive blood loss, infections, dense adhesions, birth traumas [50], all efforts should be made to improve pain relief and promote natural delivery methods.

## Limitations

Like all qualitative studies, the findings of this study cannot be generalised but can be transferred to other similar settings. The selected hospitals are both based in cities where access to drugs and specialists is relatively easier. Experiences of nurses and midwives in rural areas of the country may differ due to their relatively limited access to drugs and specialists and would need to be explored in future studies.

## Conclusions

Nurses and midwives are familiar with some non-pharmacological labour pain management techniques and frequently use them in their practice. A good number of non-pharmacologic techniques remain unknown to nurses and midwives and are subsequently not used in practice to reduce childbirth associated pain. Some of the reasons for the usage of non-pharmacologic techniques include their non-invasiveness, inexpensive nature, ease of use, safety, comfort enhancement and bonding. Nevertheless, barriers such as staff and client misconceptions about their efficacy, insufficient staff and resources prevent optimal use by nurses and midwives in Ghana. Indeed, there is support for non-pharmacological methods among nurses and midwives. However, these are mostly perceived as a second option to pharmacological methods and mainly lead to an overreliance on the latter due to its perceived superior efficacy, coupled with the other reported barriers to the utilization of non-pharmacological methods. Maternity care providers and clients alike should be educated on labour pain relief options, with emphasis on the role of non-pharmacologic techniques in complementing analgesics to provide maximal pain relief with minimal side effects. While client and staff education ought to be strengthened in this area, the identified barriers should be addressed at both institutional and national levels to improve pain management for expectant mothers. Future studies should experimentally examine the efficacy of some of these non-pharmacologic methods on labour pain relief and other birth outcomes, especially in resource-constrained settings so as to get balanced evidence as most of such studies have been conducted in developed countries.

## Additional file

Additional file 1: Interview Guide (Labour Pain Management): Interview Guide: This guided data collection for this study. (DOCX 16 kb)

## Abbreviations

RM: Rotation Midwife
SM: Staff Midwife
SNO: Senior Nursing Officer
SSM: Senior Staff Midwife
