# Supplementary material for: Nurses and midwives’ experiences of using non-pharmacological interventions for labour pain management: a qualitative study in Ghana
Source: BMC Pregnancy Childbirth. 2019 May 14;19:168. doi: 10.1186/s12884-019-2311-x (PMC6518741; doi:10.1186/s12884-019-2311-x)
Supplement: Supplementary file 1 — Interview Guide (Labour Pain Management): Interview Guide: This guided data collection for this study. (DOCX 16 kb) [file 12884_2019_2311_MOESM1_ESM.docx]

# INTERVIEW GUIDE

Study title: Nurses and midwives’ experiences of using non-pharmacological interventions for labour pain management: a qualitative study in Ghana

# PART 1

# Kindly tell me what your rank is.

# Please tell me how many years you have been working in this facility.

# Kindly enlighten me on your sole responsibilities?

# Please tell me, can labour pain be relieved?

Probes: How should labour pain be relieved? What are your experiences with relieving labour pain? Which method do you use on regular basis? Which method will you recommend to your clients and why?

# PART 2 (Labour pain management with non-pharmacological methods)

# Please tell me about the pain relief measures you are familiar with?

Probes: How did you get to know them? Kindly give me some examples?

1. What are the benefits of these methods?

# Which other pain relief methods have you heard of? How did you find out?

# What does a non-pharmacological pain management therapy mean to you?

Probe: Kindly give me some examples

# How often are non-pharmacological therapies used to manage labour pain in this facility?

# Tell me about times you have used non-pharmacological therapies to manage labour pain?

Probe: kindly relate it to its use on a client, on a relative or a friend. How did they feel after being managed with these therapies? What are your thoughts about these methods?

# Are there any barriers in providing non-pharmacological therapies to clients in your care? Kindly list them

1. Which places do you think these therapies would be readily available?

Probe:

- Traditional birth attendants
- Home delivery
- Prayer camps
- Hospital

Probe: Why will clients go to these places? What factors makes these therapies readily available at these places?

1. What can the facility do to support labour pain management?

Probe: Do you think there is a need for the hospital setting to change to meet the needs of clients in labour pain management? What are the changes you feel need to occur? What will be the main features of this new hospital setting?

1. For non-pharmacological therapies which medium of communication will you find most useful to receive information in?

Probes:

# Posters

# Leaflets

# Videos

# Audio cassettes

# PART 3 (the impact of labour pain management using non-pharmacological methods)

# Kindly tell me, do women in labour ask for pain relief measures?

Probe**:** which method do they usually ask for? Have you ever provided any of these services to these women? Are these women satisfied with these pain management therapies?

# Who should make decisions for clients on labour pain management? (Is it the client, health-worker or support person)

# What comments do you have for me? What would you like to tell me that you have thought about during this interview?

# Can I contact you later in case I have additional questions?

Thank you
